# Endophenotype‐based in silico network medicine methodology for rational design of drug combination in Alzheimer's disease

**DOI:** 10.1002/alz70859_105830

**Published:** 2025-12-26

**Authors:** Zhendong Sha, Yadi Zhou, Yuan Hou, Jeffrey L. Cummings, Feixiong Cheng

**Affiliations:** ^1^ Cleveland Clinic, Cleveland, OH USA; ^2^ Chambers‐Grundy Center for Transformative Neuroscience, Kirk Kerkorian School of Medicine, University of Nevada, Las Vegas, NV USA; ^3^ Chambers‐Grundy Center for Transformative Neuroscience, Department of Brain Health, School of Integrated Health Sciences, University of Nevada Las Vegas, Las Vegas, NV USA; ^4^ Kirk Kerkorian School of Medicine, University of Nevada Las Vegas, Las Vegas, NV USA; ^5^ Chambers‐Grundy Center for Transformative Neuroscience, Las Vegas, NV USA; ^6^ University of Nevada, Las Vegas, NV USA; ^7^ Case Western Reserve University, Cleveland, OH USA; ^8^ Cleveland Clinic Genome Center, Cleveland, OH USA

## Abstract

**Background:**

Alzheimer’s disease (AD) is a multifactorial disorder, posing significant challenges to the conventional “one drug, one disease” paradigm. Multi‐targeted therapy and complementary disease gene exposure through drug combinations have the potential to enhance effectiveness. This study leverages endophenotypes—pathobiological phenotypes underlying AD—as multifactorial disease modules to design drug combinations based on the network proximity of their targets to endophenotype modules on the protein‐protein interactome (PPI).

**Method:**

As many as eight AD endophenotypes were considered in this research. Endophenotype genes were curated from various sources, including Gene Ontology (GO), the Kyoto Encyclopedia of Genes and Genomes (KEGG), and relevant literature. These genes were specialized for AD using genome‐wide association study (GWAS) summary statistics and brain tissue quantitative trait locus (xQTL) data. Genes for each AD endophenotype were mapped to the PPI to calculate network proximity, which was used to estimate the therapeutic potential of drugs for targeting specific endophenotypes.

**Result:**

Drug combinations were prioritized using a scoring framework that evaluates common targeting and complementary exposure. Meclofenamic Acid and Amlodipine demonstrated strong therapeutic potential by targeting seven endophenotypes together. The high degree of common targeting and large network seperation between their drug targets on PPI suggest increased potential effectiveness. Memantine was found to target key AD endophenotypes, including Amyloid and Neuroinflammation, but did not target Tau. This limitation could be addressed by combining Memantine with Amlodipine. Similarly, Tofacitinib, an immunosuppressant commonly used to treat inflammatory conditions like rheumatoid arthritis, also targets Tau, suggesting that Memantine paired with Tofacitinib could also extend the endophenotype coverage of Memantine. These drug combinations are further validated using electronic health record (EHR) data, assessing their effectiveness in real‐world patient cohorts. The results demonstrated that the identified drug combinations specifically target multifactorial AD pathobiological pathways, offering valuable insights into their therapeutic potential.

**Conclusion:**

This study presents a network medicine framework for rationally designing effective drug combinations to address the complex pathobiology of AD. By integrating endophenotype‐based network medicine with large real‐world patient data analysis, this approach offers a systematic platform toward advanced drug combination design for AD and other AD‐related dementia if broadly applied.

